# A Systematic Review of Single Chinese Herbs for Alzheimer's Disease Treatment

**DOI:** 10.1093/ecam/nep136

**Published:** 2011-02-13

**Authors:** Li-Min Fu, Ju-Tzu Li

**Affiliations:** Department of Western Medicine, Southern California University of Health Sciences, Whittier, CA 90604, USA

## Abstract

The objectives here are to provide a systematic review of the current evidence concerning the use of Chinese herbs in the treatment of Alzheimer's disease (AD) and to understand their mechanisms of action with respect to the pathophysiology of the disease. AD, characterized microscopically by deposition of amyloid plaques and formation of neurofibrillary tangles in the brain, has become the most common cause of senile dementia. The limitations of western medications have led us to explore herbal medicine. In particular, many Chinese herbs have demonstrated some interesting therapeutic properties. The following databases were searched from their inception: MEDLINE (PUBMED), ALT HEALTH WATCH (EBSCO), CINAH and Cochrane Central. Only single Chinese herbs are included. Two reviewers independently extracted the data and performed quality assessment. The quality assessment of a clinical trial is based on the *Jadad* criteria. Seven Chinese herbs and six randomized controlled clinical trials were identified under the predefined criteria. *Ginkgo biloba*, Huperzine A (*Lycopodium serratum*) and Ginseng have been assessed for their clinical efficacy with limited favorable evidence. No serious adverse events were reported. Chinese herbs show promise in the treatment of AD in terms of their cognitive benefits and more importantly, their mechanisms of action that deal with the fundamental pathophysiology of the disease. However, the current evidence in support of their use is inconclusive or inadequate. Future research should place emphasis on herbs that can treat the root of the disease.

## 1. Introduction

Alzheimer's disease (AD), first described by German psychiatrist Alois Alzheimer in 1906, is a progressive neurodegenerative disease characterized by cognitive deterioration together with behavioral disturbances and declining activities of daily living [1]. AD is the most common cause of senile dementia [2] with a prevalence estimated to be 1.6% in the USA [3]. The rate is expected to increase over time as the aging population grows inasmuch as more than 80% of AD patients are 65 years or older.

Microscopically, AD is characterized by amyloid plaques and neurofibrillary tangles [4]. Amyloid plaques are dense deposits of amyloid-beta (A*β*) protein. Accumulation of aggregated amyloid fibrils, which are believed to be neuro-toxic, causes loss of neurons and synapses in the cerebral cortex and certain subcortical regions and results in gross atrophy of the affected areas [5]. However, deposition of amyloid plaques does not correlate well with neuron loss [6]. In a recent clinical trial, an experimental vaccine was found to clear the amyloid plaques, but it did not have any significant effect on dementia [7], suggesting that neurofibrillary tangles could play a more important role than amyloid plaques in the pathophysiology of AD.

The short-term memory dysfunction in AD has been related to reduction in the activity of the cholinergic neurons [8]. Among the four medications currently approved by the US FDA (Food and Drug Administration) for treating the cognitive deficiency of AD, three are acetylcholinesterase inhibitors (donepezil, galantamine and rivastigmine), which increase cholinergic activity. However, the efficacy of cholinesterase inhibitors for the treatment of AD is not supported by a high-quality large-scale randomized double-blind clinical trial [9]. Approaches based on the use of antioxidants, anti-inflammatory drugs, vitamin E and estrogen remain controversial [10].

We have identified two systematic reviews in the literature concerning the efficacy of herbal medicine for AD [11, 12]. The first systematic review included four clinical studies, among which western herbs were examined in two studies and Chinese herbs in the other two. The second systematic review published in 2008 included 16 clinical studies, among which western herbs were examined in two studies, Japanese herbs in one study and Chinese herbs in the rest of studies (13 studies). The results suggest that Chinese herbal medicine represents a promising area in the future treatment of AD. Unfortunately, Chinese herbal remedies that have undergone clinical trials are mostly in the form of herbal formulas rather than single herbs. It would be difficult to identify and validate the effective ingredients in an herbal formula that demonstrates clinical efficacy. Thus our systematic review is focused on single Chinese herbs that have effects on the development and progression of AD according to experimental and clinical evidence. This review excludes western herbs such as *Salvia officinalis* and *Melissa officinalis* that have demonstrated clinical benefits to AD patients.

## 2. Methods

The following computerized databases were searched from their inception to December 2008: MEDLINE (PUBMED), ALT HEALTH WATCH (EBSCO), CINAHL and Cochrane Central. Text word search of titles and abstracts was conducted using the following entries in various conjunction or disjunction: *Alzheimer's Disease, Chinese, herbs, and herbal medicine*. Only articles originally written in English or translated into English were considered.

Each study included in this review satisfied the following criteria: (i) the disease studied was AD and (ii) the herb studied was a Chinese herb. The exclusion criteria consisted of (i) the study was a clinical trial but it was not a randomized controlled trial (RCT), (ii) the herb studied was an herbal formula (i.e., neither a single herb nor a single herbal compound), (iii) the diseased population studied included the dementia of non-AD type (e.g., dementia due to brain ischemia or infarct) and (iv) the herb concerned was not a Chinese herb. Two reviewers independently extracted the data and performed quality assessment. The quality assessment in this review is based on the validated *Jadad* criteria, which was devised for grading the quality of RCTs [13].

## 3. Results

**Figure 1:**
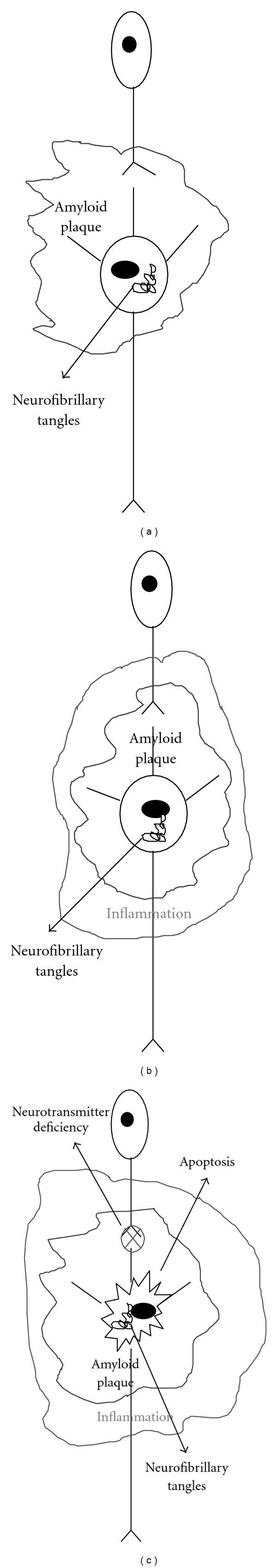
Neuronal changes in the Alzheimer's brain. In the early stage (a), there are amyloidal deposits surrounding neurons and formation of neurofibrillary tangles in the neuron. In the next stage (b), amyloidal deposits cause inflammation and damage due to oxidative stress in the brain. In the late stage (c), neurons degenerate in the process known as apoptosis and neurotransmitters dysfunction, resulting in dementia.

**Figure 2:**
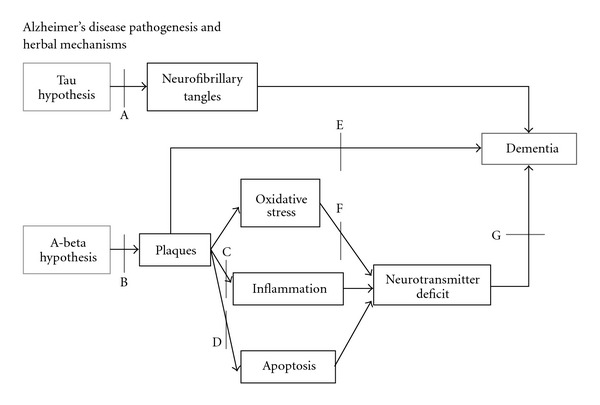
The blocking sites of herbs on the pathogenesis of Alzheimer's disease. *Ginkgo biloba*: B, D–G; Huperzine A: B, D, F, G; *Uncaria rhynchophylla*: B, E; Ginseng: B, F, G; Tenuigenin (*Polygala tenuifolta*): B; Berberine (*Coptidis rhizoma*): B, C, G; Indirubins: A.

Our review has identified seven Chinese herbs that have been studied for their potential benefits in AD treatment. Out of these seven herbs, *Ginkgo biloba*, Huperzine A and Ginseng have been assessed for clinical efficacy separately in six RCTs (Table 1). The mechanisms of action of each herb are shown in Table 2. The pathological stages of AD are drawn in Figure 1 and the pathophysiological target site of each Chinese herb is shown in Figure 2.

### 3.1. Ginkgo biloba

*Ginkgo biloba* has been used as Chinese herbal medicine to treat a variety of health disorders for centuries. Ginkgo seed is used as an astringent for problems like asthma, chronic bronchitis, spermatorrhea and leukorrhea. Ginkgo leaf is used for vascular insufficiency such as coronary heart disease. *G. biloba* exhibits several interesting properties that make it a promising herbal candidate for the treatment of AD [20]. The herb is a well-known anti-oxidant and can protect the brain from A*β*-induced oxidative damage. In addition, *G. biloba* can inhibit the A*β* fibril formation, the toxicity of A*β*-derived neurotoxins known as Amyloid-beta derived diffusible ligands (ADDLs), and A*β*-induced apoptosis. The herb also shifts the metabolism of amyloid precursor protein (APP) in favor of the non-amyloidogenic pathway. Furthermore, the herb can modulate brain cholinergic transmission, increase brain cholinergic activity, and normalize the acetylcholine receptors in the hippocampus area [10]. The *G. biloba* extract EGb 761 used in the clinical trials contained 22%–27% of flavonoids, 5%–7% terpene lactones and no more than 5 ppm alkylphenols [15].

The clinical efficacy of *G. biloba* extract EGb 761 was assessed in a clinical study conducted by Maurer et al. [14]. This study is a double-blind RCT. Twenty AD patients aged 50–80 with mild to moderate dementia were recruited. After excluding two patients, the remaining 18 patients were randomly divided into the treatment and control groups. Over a 3-month period, the treatment group received an oral daily dose of 240 mg EGb 761, whereas the control group received a placebo. The primary outcome measure was SKT (Syndrom-Kurz Test), which is a short cognitive performance test for assessing memory and attention. Secondary measures such as AD assessment scale (ADAS) and EEG were analyzed qualitatively. The study confirmed the efficacy of EGb 761, as measured by SKT, for the treatment of mildly to moderately severe AD. In addition, EEG showed some improvement in brain activity. However, the results based on the ADAS were not statistically significant, possibly due to the small sample size. The quality of this study is fair. No sufficient details were reported with respect to the procedures of randomization and blinding as well as the tracking of dropouts in the process. The validity of the study would also be weakened by the small sample size.

Schneider et al. [15] conducted a large-scale double-blind, multicenter RCT for determining the clinical efficacy of *G. biloba* extract EGb 761 in AD. Patients aged 60 years or older with mild to moderate dementia of the AD type for at least 6 months were recruited. Patients were excluded from the trial if they had any other type of dementia or central nerve system disorder or if they had taken anti-dementia medications including cholinesterase inhibitors within 6 weeks of study entry. A CT or MRI scan performed within one-year of study entry and being consistent with the diagnosis of AD was required at inclusion After initial screening, 513 patients participated in the study and they were randomly divided into three groups. Over a 26-week period, 169 patients received an oral daily dose of 120 mg, 170 patients an oral dose of 240 mg and 174 patients took a placebo. The primary outcome measures are ADAS-cog and ADCS-CGIC. The results showed no statistically significant differences between the treatment and control groups, and the efficacy of *G. biloba* on AD remained inconclusive. The high quality of this study is reflected by its large sample size, the use of strict inclusion and exclusion criteria, sound randomization and blinding techniques, and a detailed dropout report. Therefore, this study would reasonably carry more weight in its conclusion when compared with other studies.

A double-blind RCT conducted by Mazza et al. was aimed to compare *G. biloba* extract EGb 761 and a second-generation cholinesterase inhibitor, donepezil, for their efficacy in the treatment of AD [16]. Patients aged 50–80 years with a mild to moderate degree of AD dementia were recruited but patients with dementia of other etiologies were excluded. Medications with cognitive effects were prohibited. In the study, 76 patients were randomized into three groups. Over a 24-week period, 25 patients received an oral daily dose of 160 mg EGb 761; 25 patients received an oral daily dose of donepezil 5 mg; and remaining 26 patients took a placebo. The primary outcome measures were MMSE (mini-mental state examination), SKT and Clinical Global Impression (CGI). The study found that both *G. biloba* and donepezil were more effective than the placebo for improving the cognitive function in patients with mild to moderate AD according to the measures of SKT and CGI (but not of MMSE), and the differences were statistically significant; however, there was no statistical difference between *G. biloba* and donepezil based on all three measures. In addition, there were no major side effects reported. Thus, this study concluded that *G. biloba* could be a valuable alternative to cholinesterase inhibitors for the treatment of AD. The quality of the study was rated high with sound randomization and blinding procedures and a detailed dropout follow-up. The only concern is its small sample size, which may limit its external validity (i.e., generalization to other clinical settings).

### 3.2. Ginseng

Ginseng grows in Northeastern Asia. Ginseng root has been used in folk medicine in countries like China and Korea for boosting Qi (energy). It is used as an adaptogen or aphrodisiac, and can be applied to patients with low energy, immune weakness and sexual dysfunction. Research has suggested that ginseng is able to enhance psychomotor and cognitive performance, and can benefit AD by improving brain cholinergic function, reducing the level of A*β*, and repairing damaged neuronal networks [19, 28].

Ginseng as an adjuvant treatment in patients with AD was analyzed in a clinical study conducted by Heo et al. [19]. Korean Ginseng used in the study is in principle similar to Chinese ginseng. The study recruited patients aged 50 years or older with mild to moderate AD dementia, but patients with a history of psychiatric or neurological disorders were excluded. All patients included in the study had been treated with western medications used for AD for at least six months. The study design was an open-label RCT. Sixty-one patients were randomized into three groups. Fifteen patients received an oral daily dose of 9 g ginseng, 15 patients received an oral daily dose of 4.5 g ginseng and 31 patients took a placebo. The active constituents of ginseng used in this study are ginsenosides composed of Rb1 (1.96%), Rb2 (2.18%), Rc (1.47%), Rd (0.72%), Re (1.11%), Rf (0.24%), Rg1 (0.49%), Rg2 (0.13%), Rg3 (0.12%), Rh1 (0.12%) and Rh2 (0.003%). During the 12-week study period, anti-dementia medications used before the study were continued. The primary outcome measures were ADAS, Korean version of MMSE, and Clinical Dementia Rating (CDR). The high-dose ginseng group showed statistically significant improvement on the ADAS and CDR (but not MMSE) at the end of the study, when compared with the control group. However, such improvement was not observed in the low-dose ginseng group. This study was poorly designed with an insufficient description of randomization and without blinding. Furthermore, the sample size was small, and there was also the confounding effect due to concurrently administered western medications.

### 3.3. Huperzine A

Huperzine A is an alkaloid extracted from the Chinese herb *Lycopodium serratum* and used in Chinese folk medicine for the treatment of trauma, strains, swelling, schizophrenia, and so forth. [21]. The herb is a potent, reversible and selective inhibitor of acetylcholinesterase, which activity is even stronger than galantamine [12], an FDA-approved drug for the treatment of mild to moderate AD and other memory disorders. As the decline in brain cholinergic function is a major factor responsible for the memory deficit in AD, Huperzine A has emerged as a promising candidate for the treatment of AD patients. Other potentially beneficial effects, as far as AD is concerned, include modification of APP processing, protection against A*β*-induced oxidative injury and neuronal apoptosis, regulation of nerve growth factor and reduction in glutamate-induced toxicity [21, 22].

Huperzine A has shown promise for AD, as indicated by *in vitro* experiments and animal models. Clinical trials have been conducted in China to determine its efficacy on the disease, but only two studies were published in the English literature [17, 18]. On the basis of the evidence presented in the six trials conducted in China and published in the Chinese literature, a systematic review found that Huperzine A had some beneficial effects and no serious adverse effects for patients with AD [29]. Among the four trials considered in one analysis based on the measure of MMSE, two trials produced statistically significant results in favor of Huperzine A over placebo, two showed no statistical difference between the herb and placebo, but combining evidence quantitatively yielded a positive result. In terms of the measure of ADAS-cog, a single trials with more than 200 patients found that Huperzine A was superior to placebo statistically. Taken together, only one study [18] was of adequate quality and sample size, and the overall evidence was inadequate to support its clinical use at present. More high-quality large-scale RCTs are needed for further determination of its efficacy.

### 3.4. Other Promising Herbs

The Chinese herb *Uncaria rhynchophylla* is used for the treatment of headache, convulsion and hypertension in Chinese medicine. It is a potentially novel therapeutic agent to prevent or treat AD because it has potent inhibitory effects on fibril formation of A*β* and can also destabilize preformed A*β* fibrils [23].

Tenuigenin is a compound extracted from the Chinese herb *Polygala tenuifolta*, which exhibits the property of soothing the mind and is indicated for insomnia, mental confusion and disorientation in Chinese medicine. Research has found that tenuigenin can inhibit the secretion of A*β* in cultured cells, which may explain its ability to improve cholinergic function degraded through A*β* in rat models [24].

Berberine is an alkaloid isolated from the Chinese herb *Coptidis rhizome*. The herb has been used in folk medicine because of its antidiarrheal, antimicrobial and anti-inflammatory properties. Research findings indicate that berberine can reduce A*β* secretion by altering APP processing in a way to shift from the amyloidogenic to non-amyloidogenic pathway [26].

Indirubin is an active ingredient of a Chinese herbal formula, Danggui Longhui Wan, used in the treatment of chronic diseases such as leukemia. Its potential value in the treatment of AD patients is attributable to its property of inhibiting two protein kinases involved in abnormal tau phosphorylation in AD [27].

## 4. Discussion

AD is a progressive neurodegenerative disorder. In light of the limited effectiveness and potential side effects of the current AD medications, there is increasing interest in using herbal medicine as an alternative or complementary therapy for AD. Chinese herbs are particularly promising, as they dominate the herbs that have undergone clinical trials. However, these Chinese herbs, as included in previous systematic reviews [11, 12], are mostly in the form of formulas rather than single herbs. From the scientific standpoint, it is important to understand the pharmacological property of a single herb and its active chemical ingredients. However, formula validation leaves no clue as to what the active herbal components are in the formula, how they interact, and which herbs should be blamed for an adverse reaction if it happens. The present review distinguishes from other related reviews by focusing on single Chinese herbs and examining their effects on the pathophysiology of AD.

The results of the three RCTs assessing the efficacy of *G. biloba* in AD are mixed. More specifically, the studies conducted by Maurer et al. [14] and Mazza et al. [16] found statistically significant differences in favor of the herbal intervention over the placebo control, whereas the study conducted by Schneider et al. [15] showed no significance differences between the herbal and control groups. However, the Schneider's study would have more impact than the other two studies in terms of the sample size and quality of study. Further observation indicated that the inconsistency among these studies could be due to the difference in the chosen outcome measures. Two studies [14, 16] with positive conclusions for the herbal intervention used the SKT test, while the third study [15] with a negative conclusion used the ADAS-cog scale. The SKT test is designed to assess the deficits of memory and attention, while ADAS-cog measures the disturbances of memory, language, praxis, attention and other cognitive abilities that are often referred to as the core symptoms of AD [30]. ADAS-cog involves more difficult cognitive tasks than SKT in our opinion. Accordingly, the evidence based on the three studies, if viewed together consistently, would indicate that *G. biloba* improves the cognitive function of AD patients at the level measurable by SKT but not to the level measurable by ADAS-cog. The results illustrate the importance in the choice of the primary outcome measure in the study design. The final interpretation of a trial outcome has to take this factor into account. The efficacy and safety of *G. biloba* in AD will be further determined by a number of ongoing RCTs registered in the National Institute of Health registry of clinical trials.

Currently FDA-approved medications for AD such as acetylcholinesterase inhibitors improve cognitive function but do not treat the cause of the disease characterized by the deposition of amyloid plaques and formation of neurofibrillary tangles. On the other hand, the Chinese herbs, as analyzed in the present review, addressed the fundamental mechanisms of AD one way or another (Table 2 and Figure 2) in contrast to western herbs such as *S. officinalis* and *M. officinalis*.

**Figure 3:**
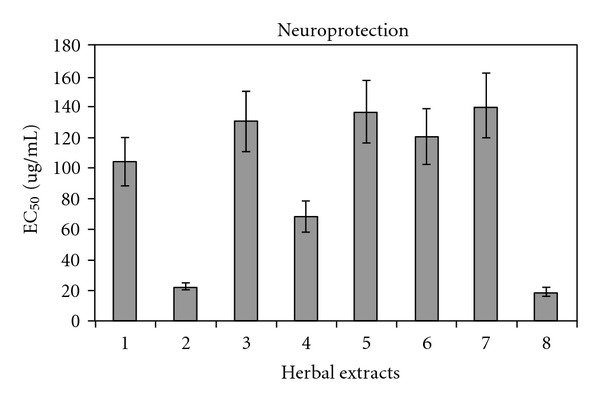
Neuroprotectivity against amyloid protein based on the study by Kim et al. [31]. 1, *Cinnamomum cassia*; 2, *Curcuma aromatica*; 3, *Gastrodia elata*; 4, *G. biloba*; 5, *Inula helenium*; 6, Polygonatum sp.; 7, *Scutellaria baicalensis*; 8, *Zingiber officinale*; EC_50_: Concentration to achieve 50% cell (primary neuron) viability.

Neuroprotectivity from amyloid insults on neurons has been assessed for various herbs (Figure 3). As research indicates that the deposition of amyloid plaques alone does not account for the entire degenerative process of AD [6], the theory based on neurofibrillary tangles may offer a more plausible explanation about its pathophysiology. To date, it is not clear yet whether and to what extent herbal medicine can reverse the progressive course of the disease by resolving amyloid plaques or neurofibrillary tangles, despite that some cognitive benefits from clinical studies have been demonstrated. The efficacy of medical or herbal treatment was assessed on mild to moderate AD patients in the clinical trials conducted to date. It makes sense to test herbal neuroprotectivity on mild AD cases for stopping disease progression in future clinical trials.

## 5. Conclusion

Chinese herbs show promise in AD treatment because of their cognitive benefits and more importantly, their mechanisms of action with respect to the fundamental pathophysiology of the disease. However, most of the clinical studies in previous reviews used herbal formulas rather than single herbs. Our review here has identified seven single Chinese herbs with potential therapeutic effects for AD. Among them, *G. biloba*, Huperzine A (*Lycopodium serratum*), and Ginseng have been assessed for their clinical efficacy separately in six randomized controlled clinical trials. Nonetheless, our review here has found that the current clinical evidence in support of the use of Chinese herbs in AD is still inconclusive or inadequate. However, no serious adverse events were reported. The data from other high-quality, large-sample RCTs currently underway are expected to provide further evidence for the clinical efficacy of Chinese herbal medicine in AD treatment. Moreover, the future direction should emphasize the trial of new herbs that are potentially effective in treating the root of the disease.

## Funding

Southern California University of Health Sciences.

## Figures and Tables

**Table 1:** The study design, herbal intervention, outcome measures, and results of RCTs concerning single Chinese herbs in the treatment of AD.

Study	Design	Herb name	Quality	*Rx N*	*Cx N*	Outcome measures^a^	Side effects	Results
Maurer et al. [14]	Randomized, double-blind, placebo-controlled, parallel	*G. biloba* (EGb761) 80 mg tid for 3 months	3	10	10	SKT	No adverse events	*Rx*> Placebo (SKT) (*P* < .05)

Schneider et al. [15]	Randomized, double-blind, placebo-controlled, parallel, multi-center	*G. biloba* (EGb761) 120 mg or 240 mg per day for 26 weeks	4	169 (120 mg) 170 (240 mg)	174	ADAS-cog, ADCS-CGIC	Related serious adverse events (1% in 120 mg Gp and 2% in 240 mg Gp)	*Rx* = Placebo

Mazza et al. [16]	Randomized, double-blind, placebo-controlled, parallel	*G. biloba* (EGb761) 160 mg per day for 24 weeks	5	25	25^b^, 26^c^	SKT MMSE CGI	No adverse events	(1) *Rx* = Donepezil(2) *Rx*> Placebo (SKT, CGI) (*P* < .001)(3) *Rx* = Placebo (MMSE)

Xu et al. [17]	Randomized, double-blind, placebo-controlled, parallel, multi-center	Huperzine A 0.2 mg b.i.d. for 8 weeks	3	50	53	Wechsler, Hasegawa, MMSE	No severe side effects	*Rx*> Placebo (*P* < .05)

Zhang et al. [18]	Randomized, double-blind, placebo-controlled, parallel, multi-center	Huperzine A 0.4 mg per day for 12 weeks	4	100	102	ADAS MMSE	Ankle edema, insomnia (3%)	*Rx*> Placebo (*P* < .001)

Heo et al. [19]	Randomized, non-blinding, controlled, parallel	Ginseng 4.5 g or 9.0 g per day for 12 weeks	1	15 (9 g)15 (4.5 g),	31	ADAS, MMSE, CDR	Nausea, fever (13%)	(1) *Rx* (9 g) >*Cx* (ADAS-cog, CDR) (*P* < .05)(2) *Rx* = *Cx* (MMSE)

*Rx*, Herbal treatment; *Cx*: Control intervention. *Rx*  >  *Cx*: The treatment is more effective than the control intervention. *Rx* = *Cx*: No statistically significant difference between the treatment and control interventions.

^a^Primary outcome measure; ^b^Donepezil 5 mg per day; ^c^Placebo.

**Table 2:** The mechanisms of action of single Chinese herbs in the treatment of AD.

Herb	Mechanisms of actions
	(i) Increase in cholinergic neuron function,
	(ii) Protection against the A*β* protein induced oxidative damages (degrading hydrogen peroxide, preventing lipid from oxidation and trapping reactive oxygen species),
*G. biloba* [20]	(iii) Prevention of A*β* protein induced fibrillogenesis as well as the formation ADDLs,
	(iv) Inhibition of cholesterol-induced overproduction of APP,
	(v) Anti-apoptosis (opposing mitochondria-initiated apoptosis, downgrading caspase-12, upgrading BCL_2_),
	(vi) Regulation of gene expression

	(i) Protection against the A*β* protein and hydrogen peroxide induced oxidative damages
	(ii) Anti-apoptosis (regulating gene expression: Bcl-2, Bax, P53 and caspase-3),
	(iii) Modulating secretary APP and protein kinase C-*α*,
Huperzine A [21, 22]	(iv) Protection against hypoxia, ischemia and glutamate induced brain injury and cytotoxiicity,
	(v) Antagonizing effects on NMDA (N-methyl-_D_-aspartate) receptors,
	(vi) Regulation of the expression and secretion of nerve growth factor and its signaling

*Uncaria rhynchophylla* [23]	(i) Inhibition of aggregation of A*β* protein,
	(ii) Destabilization of preformed A*β* neurofibrills

	(i) Increase in the uptake of choline in central nervous system,
	(ii) Release of acetylcholine from hippocampus,
Ginseng [19]	(iii) Increase in choline acetyltransferase,
	(iv) Protection against the A*β* protein induced neurotoxic effects,
	(v) Repair of A*β*-damaged neuron networks,
	(vi) Reducing the level of A*β*

Tenuigenin (*P. tenuifolta*) [24]	(i) Decrease of the secretion of A*β* protein via BACE1 (*β*-secretase) inhibition

	(i) Alteration of the processing of amyloidal precursor protein,
Berberine (*C. rhizoma*) [25, 26]	(ii) Decrease of the secretion of A*β* protein,
	(iii) Acetylcholinesterase inhibitory activity

Indirubins [27]	(i) Inhibition of abnormal tau phosphorylation by inhibiting glycogen synthase kinase-3 beta and CDK5/p25
